# Intron-Associated Mitogenome Size Variation and Gene-Specific Stop Codon Absence in the Lichen-Forming Genus *Ramalina*

**DOI:** 10.3390/jof12100729

**Published:** 2026-09-30

**Authors:** Seung-Yoon Oh, Yehyeon Cha, Jung-Jae Woo, Chang Wan Seo, Yong Sang Kim

**Affiliations:** 1 School of AI-Integrated Biological Sciences, Changwon National University, Changwon 51140, Republic of Korea; 2 Department of Biology and Microbiology, Changwon National University, Changwon 51140, Republic of Korea; 3 Gyeongnam Bio and Anti-Aging Core Facility, Changwon National University, Changwon 51140, Republic of Korea; 4 Global Institute for Advanced Nanoscience & Technology, Changwon National University, Changwon 51140, Republic of Korea; 5 Water and Environmental Research Institute of the Western Pacific, University of Guam, Mangilao, GU 96923, USA; ysk9785@nyu.edu; 6 Department of Civil, Urban, and Environmental Engineering, Tandon School of Engineering, New York University, 6 Metrotech Center, Brooklyn, NY 11201, USA

**Keywords:** group I introns, Lecanoromycetes, lichens, mitochondrial genomes, Ramalinaceae

## Abstract

Mitochondrial genomes of lichen-forming fungi provide a useful system for examining genome evolution, yet the fruticose lichen genus *Ramalina* has not been studied comparatively. We newly sequenced three *Ramalina* mitogenomes and reconstructed one from published data, then compared these with six additional public mitogenomes to obtain a 10-species dataset. Nine other Ramalinaceae mitogenomes were used as a reference panel. Within *Ramalina*, core gene content and gene order are highly conserved, whereas genome size varies 1.83-fold and is strongly associated with intron content and homing endonuclease abundance. Introns repeatedly occupy homologous insertion sites across the genus. Four genes, *cox1*, *nad3*, *nad4*, and *nad5*, recurrently lack canonical stop codons at conserved terminal positions, while *cob* consistently lacks a stop immediately after the conserved ENSLIELA motif. These states are supported by multiple assembly approaches and reanalysis of public reads, while the affected genes show patterns consistent with purifying selection. These results show that *Ramalina* mitogenomes combine conserved core architecture with intron-associated size variation and recurrent changes in mitochondrial gene-terminal signals.

## 1. Introduction

Fungal mitochondrial genomes are relatively gene-poor, but their sizes can vary substantially even among closely related species [1]. Much of this variation reflects the gain and loss of self-splicing introns, the homing endonuclease genes (HEGs) carried by many introns, and changes in intergenic sequence [2,3]. Mitochondrial introns can also have direct physiological consequences through their effects on gene expression and genome stability [4]. In several plant-pathogenic fungi, the presence of an intron immediately downstream of codon 143 of *cob* limits the emergence of the G143A substitution associated with QoI fungicide resistance [5]. In *Podospora anserina*, a mobile intron within cox1 contributes to mitochondrial genome rearrangement and is closely associated with organismal senescence [6].

Lichen-forming fungi have received particular attention because the symbiotic lifestyle was proposed to influence mitochondrial gene content [7], although that interpretation was later questioned [8]. Repeated intron gain and loss have also been documented across lichenized lineages [9]. Comparative mitogenomic studies are now available for several lichen genera, including *Cladonia* [10], *Peltigera* [11], and *Xanthoparmelia* [12]. These studies show that intron content can be a major contributor to mitogenome size variation, while core gene content and gene order are often relatively conserved at the genus level. Variation can also occur at mitochondrial gene termini, where canonical stop codons are not always present at conserved C-terminal positions. For example, *nad3* of the lichen-forming fungus *Umbilicaria pustulata* lacks a recognizable canonical stop codon [13].

*Ramalina* Ach. is a subcosmopolitan genus of fruticose lichens with roughly 230 recognized species and is the type genus of Ramalinaceae [14,15]. Despite the diversity of the genus, no comparative analysis of *Ramalina* mitogenomes has been published. Public mitochondrial sequences are available from several independent projects, including the annotated RefSeq genome of *R. intermedia*, three assemblies recovered through large-scale mining of fungal sequencing data [16], a Darwin Tree of Life assembly of *R. siliquosa* [17], and a public mitochondrial record of *R. sinensis*. Notably, *nad4* and *cob* are annotated as 3′-partial in the *R. intermedia* RefSeq mitogenome (NC_044483.1). These records have not previously been analyzed together under a uniform annotation framework, and it remains unclear whether unusual terminal states recur across *Ramalina* or are preferentially associated with particular genes.

Here, we characterize four *Ramalina* mitogenomes, including three assembled from our Illumina data and one reconstructed from the published *R. farinacea* genome assembly and long reads from the same isolate [18]. Together with six publicly available *Ramalina* mitogenomes, these sequences form a primary 10-species dataset for comparative analyses within the genus. Nine additional Ramalinaceae mitogenomes were reannotated using the same workflow and used as a secondary reference panel. We examined genome-size variation, intron distribution and HEG content, and the conservation of canonical stop codons at homologous C-terminal positions of mitochondrial protein-coding genes.

## 2. Materials and Methods

### 2.1. Specimens, Sequencing and Mitogenome Assembly

Three *Ramalina* samples were used for mitogenome sequencing. *Ramalina pacifica* Asahina was collected from the trunk of a coconut palm (*Cocos nucifera* L.) on the campus of the University of Guam, Guam, USA (13°25′52.41″ N, 144°48′15.21″ E). *Ramalina yasudae* Räsänen was collected from a rock surface in a mixed forest in Ulju-gun, South Korea (35°23′53.89″ N, 129°13′56.74″ E). An axenic mycobiont culture of *Ramalina intestiniformis* Kashiw. & K.H. Moon was obtained from a thallus collected from a coastal rock surface in Yeoseodo, Wando-gun, South Korea (33°59′5.55″ N, 126°55′19.36″ E). The strain was maintained on malt-yeast agar. Preliminary identification was based on morphological characteristics, and species identity was further assessed using ITS sequence data. Genomic DNA was extracted from thallus tissue or cultured mycelium using a modified CTAB method [19]. Total DNA from the two thallus samples and the cultured mycobiont was sequenced using Illumina paired-end sequencing on a NovaSeq platform (Macrogen, Seoul, Republic of Korea). Raw reads were quality-filtered with Trimmomatic v0.39 [20], including adapter removal, sliding-window quality trimming (4:15), and a minimum read length of 36 bp.

Mitochondrial contigs were assembled with GetOrganelle v1.7.7.1 [21] and independently checked with NOVOPlasty v4.3.1 [22] using a conserved *cox1* fragment as the seed. Both approaches recovered identical terminal regions for the stop-codon analysis, and circularity was confirmed by read support across the assembly origin. Potential non-target organellar sequences were screened against reference Ascomycota mitochondrial proteins and *Trebouxia* organelle sequences [23].

A fourth mitogenome, from *R. farinacea* isolate LIQ254RAFAR, was reconstructed using the published mitochondrial contig JAPUFD010000030.1 (GCA_029948375.1) and same-isolate Oxford Nanopore reads (SRR22733898) [18]. The reads were reassembled with Flye v2.9.6-b1802 [24], and the reassembly was used to restore a missing segment spanning the *cox1*–*nad4* junction and to correct a homopolymer-associated frameshift in nad2 based on read support and conservation of the coding frame. The resulting 29,826 bp circular mitogenome was used in subsequent analyses.

Four public *Ramalina* datasets with available raw reads were independently reassembled to validate the corresponding database sequences (Supplementary Table S1). These assemblies were used only for validation and were not treated as independent taxa in the phylogenetic or codon-based analyses.

### 2.2. Comparative Datasets, Uniform Annotation and Intron Analysis

The primary comparative dataset comprised the four mitogenomes characterized here and six public *Ramalina* genomes: *R. intermedia* (NC_044483.1), *R. americana* (BK074020.1), *R. culbersoniorum* (BK074021.1), *R. petrina* (BK074022.1), *R. siliquosa* (OZ244615.1), and *R. sinensis* (OP271861.1) (10 species; Table 1). Nine additional Ramalinaceae mitogenomes formed a secondary reference panel (Supplementary Table S1). All 19 sequences were reannotated with the same pipeline. Protein-coding genes were predicted with MFannot v.1.36 [25] under NCBI genetic code 4 and with miniprot v0.18 [26] using a Ramalinaceae protein reference set. tRNAs were predicted with tRNAscan-SE v2.0.13 [27], and rRNAs were identified by BLASTN v2.17.0+ searches against reference rRNA sequences. MFannot v1.36 was used to annotate introns, intron subtypes, and intronic ORFs. Homing endonuclease genes were identified independently by extracting genome-wide six-frame stop-to-stop ORFs of ≥80 amino acids under NCBI genetic code 4and screening them with HMMER v3.4 (E ≤ 1 × 10^−5^) [28] against Pfam-A v38.2 [29]. ORFs containing LAGLIDADG (PF00961) or GIY-YIG (PF01541) domains were retained when they overlapped MFannot-predicted intronic ORFs and were subsequently curated for their initiation coordinates. The three newly sequenced genomes were manually curated by comparison with *R. intermedia* proteins. Exon boundaries, initiation codons, and conserved C-terminal regions were checked under NCBI genetic code 4, and tRNAs were named by anticodon. Alignment-derived terminal mini-exons lacking intron support were excluded.

Introns were defined as gaps of at least 100 bp between consecutive exons of retained protein-coding or rRNA gene models. Gaps beyond a conserved C-terminus or overlapping an exon of another gene were excluded unless independently supported by occurrence in at least two genomes or by an MFannot subtype assignment. Insertion sites were projected onto the codon coordinates of the *Bacidia sorediata* (MK294991.1) reference proteins by aligning exon-flanking sequences. Genus-level summaries were based on the 10 *Ramalina* genomes, and associations of genome size with intron number, total intron length, and HEG number were evaluated using Spearman rank correlations and linear regression. The Ramalinaceae reference panel was used only to assess whether homologous insertion positions also occurred outside *Ramalina*.

### 2.3. Translation-Termination Signals, Codon Usage and Repeat Analysis

For each core gene, a homologous reference stop position was defined from a genome retaining a canonical stop when available; this positional reference was not treated as an inferred ancestral state. *R. intermedia* was the primary reference, *Niebla homalea* was used for *nad4*, and alternative nearby termini were scored for *nad3* and *nad5* (Supplementary Table S4). The C-terminal 60 residues of each reference were aligned to translations extending 900 bp downstream, and the codon at the aligned terminus and distance to the first downstream in-frame TAA or TAG were recorded. States were classified as retained, proximal downstream stop (1–49 codons), or extended stop-free (no stop within 50 codons). For *cob*, the conserved ENSLIELA motif (up to two mismatches) was used as the anchor; because no sampled genome had an immediate stop, the following codon was reported descriptively.

Relative synonymous codon usage (RSCU) was calculated from the 15 core protein-coding genes under NCBI genetic code 4, using coding regions truncated at the conserved terminal positions. *Ramalina* was treated as the primary dataset, and the additional Ramalinaceae genomes were used as a reference comparison (Supplementary Table S3). Simple sequence repeats (SSRs; motif sizes 1–6 bp) were identified using MISA-style minimum repeat numbers: 10 for mononucleotide, 6 for dinucleotide, and 5 for tri- to hexanucleotide motifs [30]. Longer tandem repeats were defined as exact repeats with unit sizes of 7–60 bp, at least two copies, and a total length of ≥20 bp. Dispersed repeats were identified as maximal exact forward or palindromic repeat pairs of ≥30 bp using REPuter-style criteria [31] (Supplementary Table S6).

### 2.4. Phylogenetic Analysis and Selection

Fourteen protein-coding genes were used for phylogenetic analysis; *rps3* was excluded because of its low conservation. Coding regions were truncated at the reference terminal positions and aligned as amino acids with MAFFT v7.520 (L-INS-i) [32], trimmed with trimAl v1.4.1 (automated1) [33], and concatenated into a matrix. Models containing internal stop codons or differing from the *Ramalina* median length by more than 20% were excluded, removing *cox1* of *Biatora appalachensis*, *nad5* of *B. longispora*, and *nad6* of *Micareopsis irriguata*. A maximum-likelihood tree was inferred with IQ-TREE v2.2.2.9 [34] using a partitioned ModelFinder scheme [35], 1000 ultrafast bootstrap replicates [36], and 1000 SH-aLRT replicates. Selection was assessed with FEL in HyPhy 2.5.101 [37,38], and pairwise dN and dS were calculated using the Nei-Gojobori method with Jukes-Cantor correction [39]. Codon alignments were obtained by back-translating the trimmed amino-acid alignments; FEL sites were called at *p* ≤ 0.1, and pairwise dN/dS was averaged over pairs with 0 < dS ≤ 1.5.

## 3. Results

### 3.1. Mitogenome Characteristics and Intron-Associated Variation

**Figure 1:**
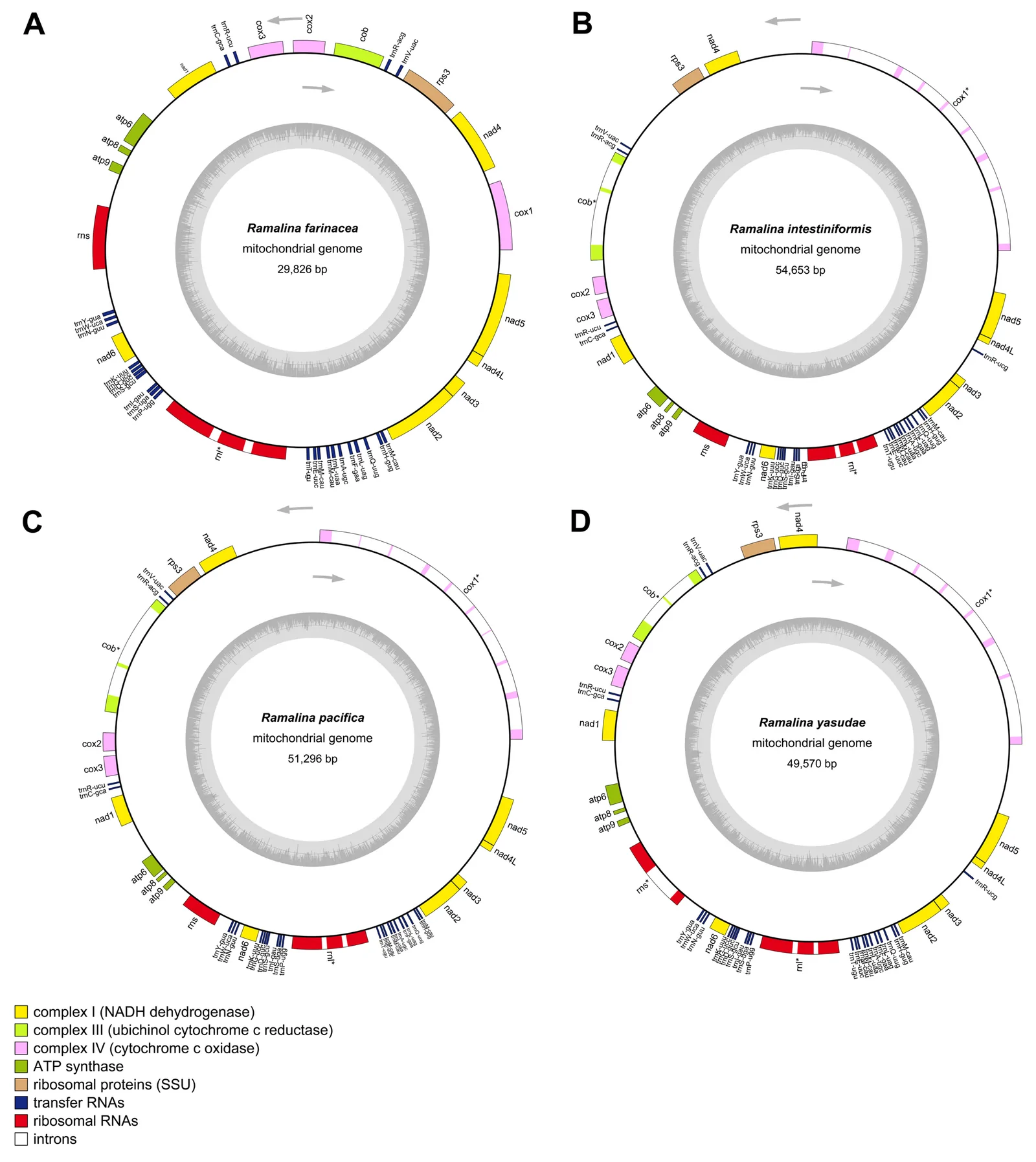
Circular maps of the four *Ramalina* mitogenomes characterized in this study. Panels represent (**A**) *Ramalina farinacea*, (**B**) *R. intestiniformis*, (**C**) *R. pacifica*, and (**D**) *R. yasudae*. The outer ring shows protein-coding genes, rRNA genes, and tRNA genes. White segments indicate introns, and asterisks indicate intron-containing genes. The inner ring shows intron-encoded HEG ORFs.

**Figure 2:**
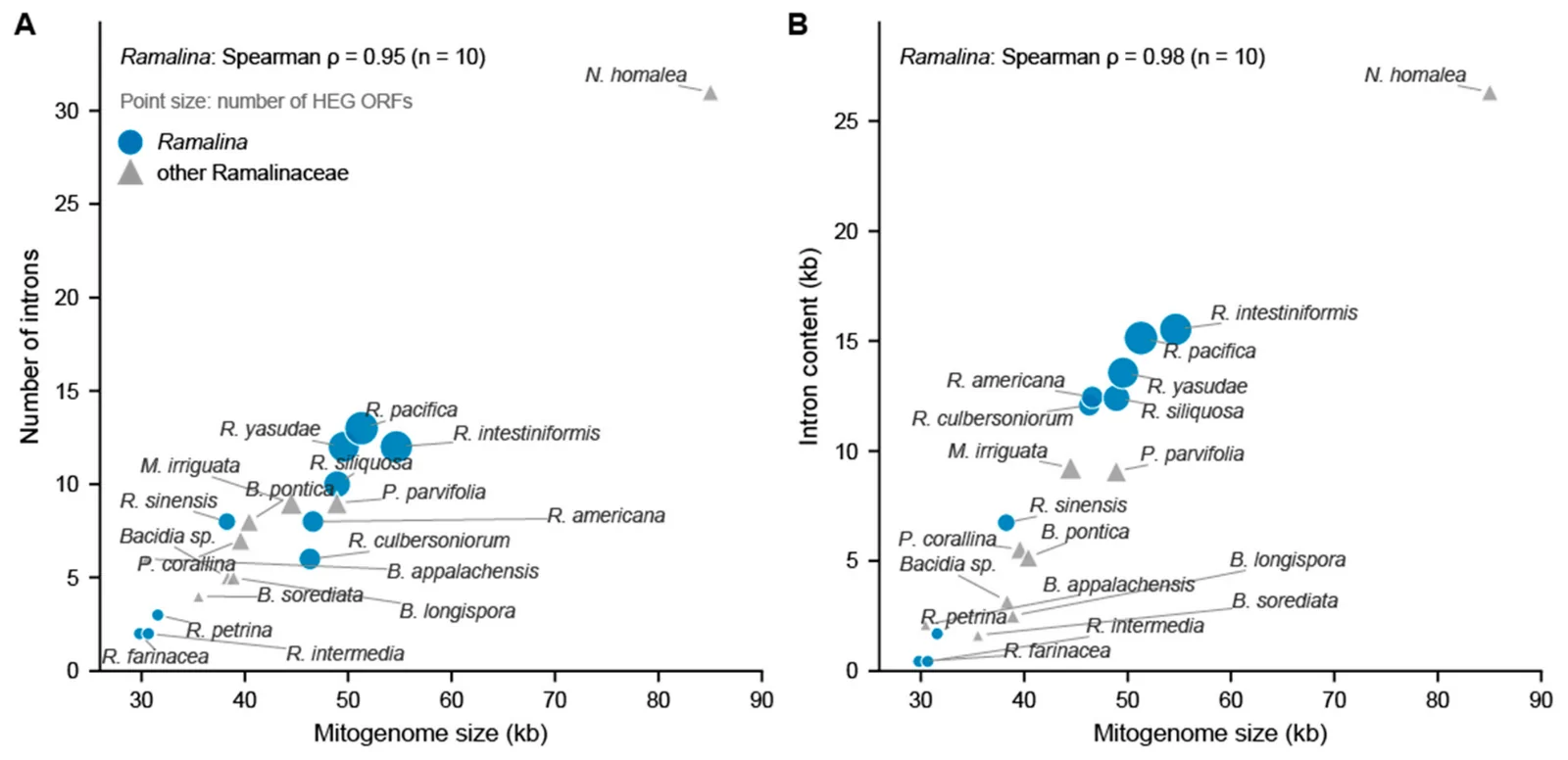
Mitogenome size in relation to (**A**) intron number and (**B**) total intron content. Spearman rank correlations are calculated from the 10 *Ramalina* species; the additional Ramalinaceae genomes are shown as reference points. Point size is proportional to the number of intron-encoded homing endonuclease ORFs.

The three newly sequenced mitogenomes are circular molecules of 49,570 bp in *R. yasudae*, 51,296 bp in *R. pacifica*, and 54,653 bp in *R. intestiniformis*; the reconstructed *R. farinacea* mitogenome is 29,826 bp. The 10 available *Ramalina* mitogenomes therefore span 29,826–54,653 bp, a 1.83-fold range (Table 1; Figure 1), and share the same 15 core protein-coding genes and two rRNA genes; their tRNA complements vary from 25 to 26 genes. Genome size is strongly associated with intron content within *Ramalina*. Intron number ranges from 2 to 13, total intron content from 436 to 15,533 bp, and HEG number from 0 to 12 (Figure 2; Table 1). Genome size correlates with intron number (Spearman rho = 0.95), total intron content (rho = 0.98), and HEG number (rho = 0.97; all *p* < 0.001). The three smallest genomes contain only two or three introns and no recognizable HEGs, whereas the six genomes larger than 46 kb contain 6 to 13 introns and 4 to 12 HEGs. Linear regression indicates that approximately 65% of the increase in mitogenome size across the ten species is associated with increased intron content (R^2^ = 0.99); the smallest and largest genomes similarly differ by 24.8 kb overall and 15.1 kb in intron content. Given the conserved core gene content and uniformly low repeat content (Supplementary Table S6), most of the remaining size variation is likely intergenic.

Protein-coding introns repeatedly occupy homologous positions within *Ramalina* (Supplementary Table S2). Eleven sites occur in at least three species; *cox1* codon 347 occurs in six species, and codons 66, 101, and 241 occur in five. The intron-rich *R. yasudae*, *R. pacifica*, and *R. intestiniformis* largely use positions shared with other *Ramalina* genomes. Most *cox1* introns in the newly sequenced genomes are group IB, and all but one of their 30 protein-coding introns (the second *cob* intron of *R. yasudae*) contains a recognizable LAGLIDADG or GIY-YIG domain (Supplementary Table S2). This recurrence and HEG occupancy are consistent with retained homing potential but do not establish intron age or current mobility.

### 3.2. Gene Order, Base Composition, Codon Usage and Repeats

**Figure 3:**
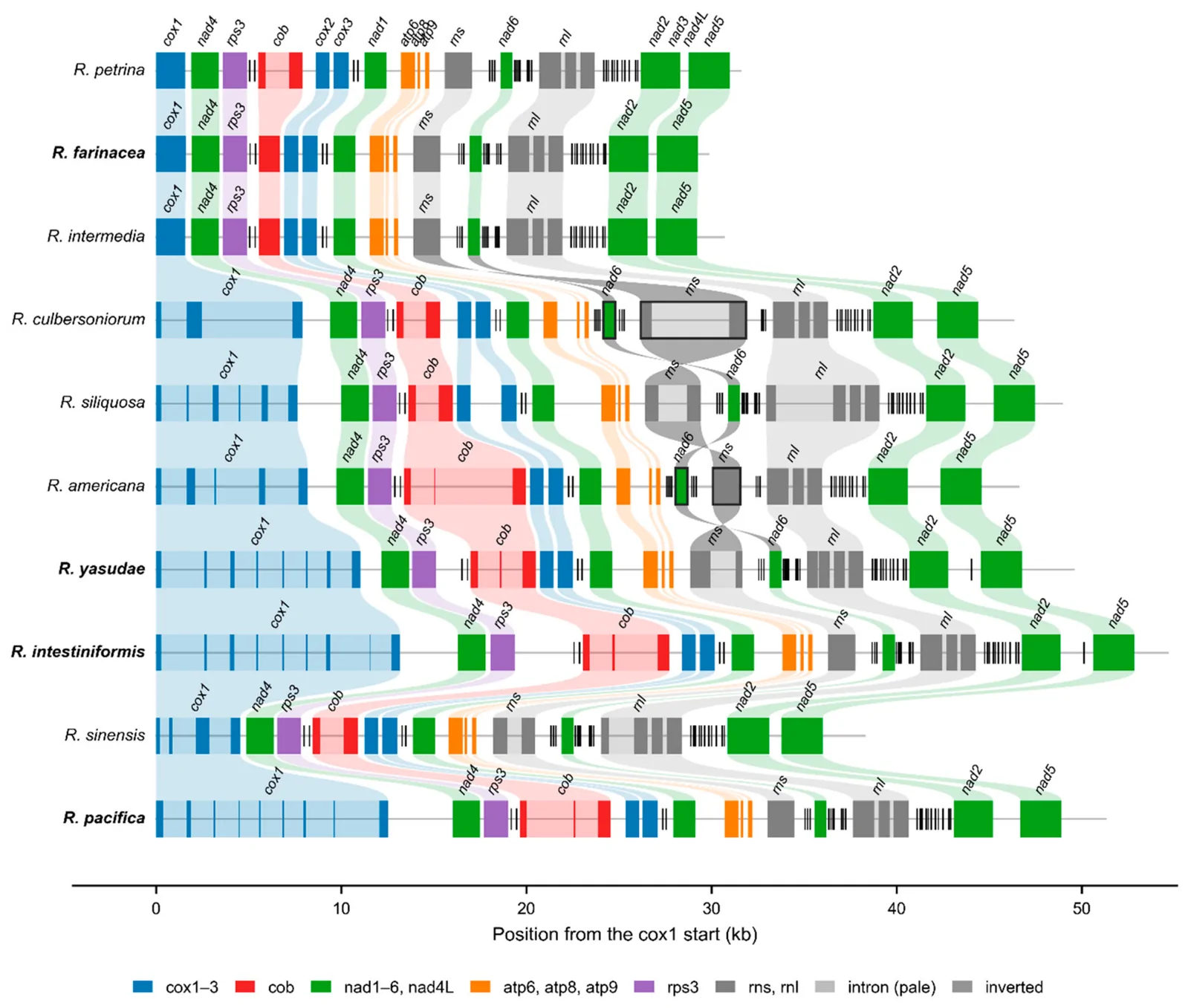
Synteny and gene-order comparison of *Ramalina* mitogenomes. Synteny of the 15 core protein-coding genes and two rRNA genes across the 10 *Ramalina* mitogenomes. Exons are shown in solid fill and introns in pale fill; ticks indicate tRNA genes, and dark ribbons and outlined boxes indicate strand inversions. Genomes characterized in this study are in bold.

Gene order is highly conserved across the 10 *Ramalina* mitogenomes. The main exception is an inversion containing *nad6*, *rns*, and seven tRNA genes in *R. americana* and *R. culbersoniorum* (Figure 3), which was reproduced in the independent *R. americana* reassembly. This gene order is not unique to *Ramalina*: *Niebla homalea* shares the same arrangement, whereas the other sampled Ramalinaceae genera differ mainly in the position of *cox3*, with additional rearrangements in *Micareopsis* and *Bacidia* (Supplementary Figure S1).

*Ramalina* mitogenomes are AT-rich, with AT contents of approximately 68.1 to 69.7%. RSCU profiles are highly similar among the 10 species and are biased toward A- or T-ending codons; TGA is used more frequently than TGG for tryptophan under NCBI genetic code 4 (Supplementary Table S3). Repeat content is low, and dispersed repeats account for less than 2% of genome length in all *Ramalina* genomes examined (Supplementary Table S6).

### 3.3. Mitochondrial Phylogeny and Recurrent Stop-Codon Absence

**Figure 4:**
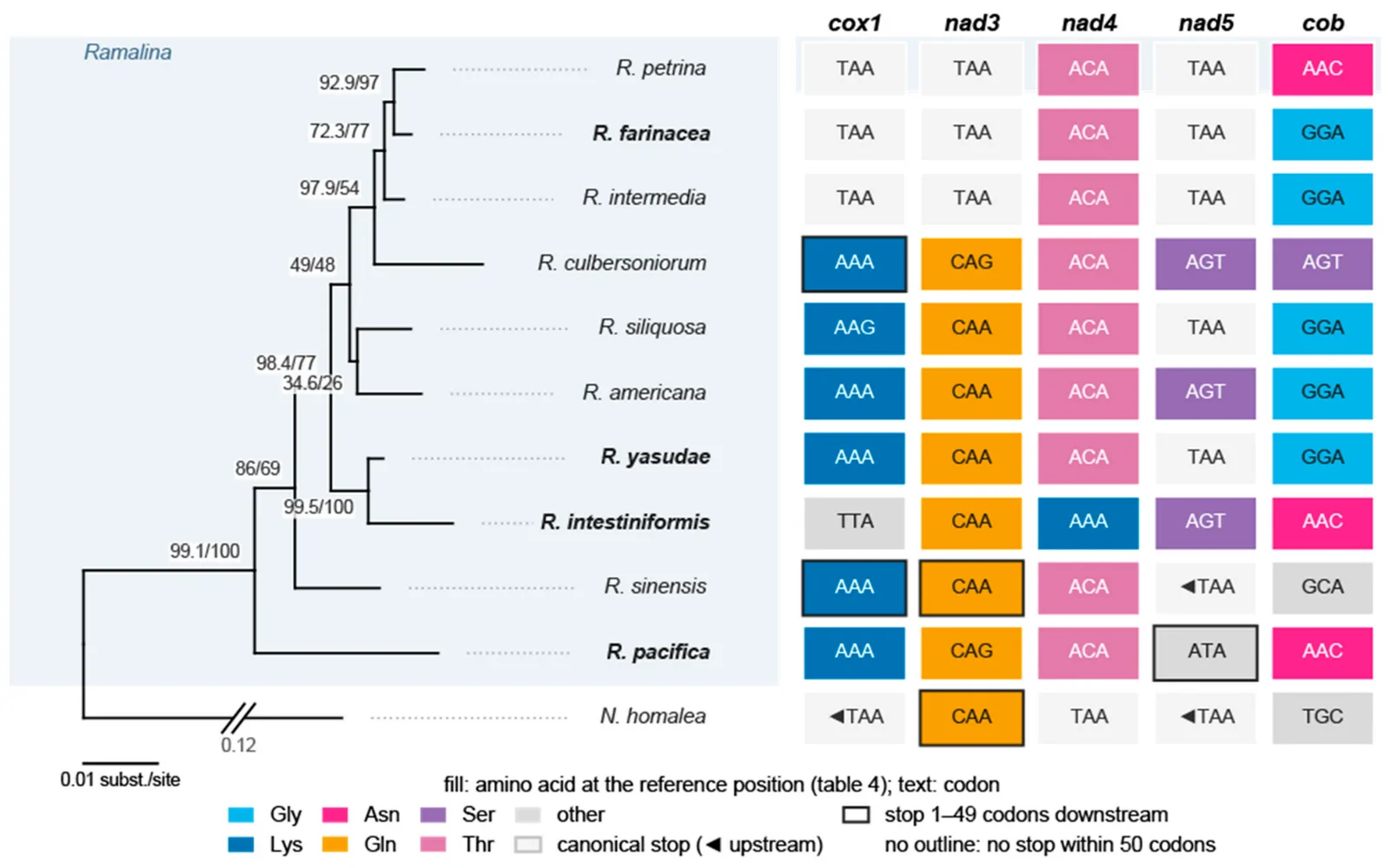
Mitochondrial phylogeny and stop-codon states of five affected genes in *Ramalina*. The 10 *Ramalina* species and *N. homalea* form a subtree of the 19-taxon maximum-likelihood tree; branch lengths and SH-aLRT/ultrafast-bootstrap supports are from the full analysis, and the *N. homalea* branch is shortened (true length, 0.12 substitutions per site). Species names in bold indicate the four genomes characterized in this study. Tip-aligned cells show the terminal states of *cox1*, *nad3*, *nad4*, *nad5*, and *cob*. Cell text indicates the codon at the reference stop position or, for *cob*, after the conserved ENSLIELA motif. Unshaded cells indicate canonical stops; ◂ denotes stops 2–4 codons upstream. Outlined cells have an in-frame stop 1–49 codons downstream; unoutlined cells have none within 50 codons. Fill colors indicate amino acids shared by at least two genomes within a gene; other codons are grey. Reference-panel states are shown in Supplementary Figure S2 and Table S4.

The 14-gene mitochondrial analysis recovers *Ramalina* as monophyletic with strong support, although most relationships within the genus are weakly resolved (Figure 4). The analysis supports the generic placement of the three newly sequenced specimens but was not used to infer family-level relationships. The *nad4* terminal state is shared by all sampled *Ramalina* species, whereas *cox1*, *nad3*, and *nad5* vary among species.

Across the 15 core protein-coding genes, 10 retain TAA or TAG at the conserved terminal position in all 10 *Ramalina* species. Four genes, *cox1*, *nad3*, *nad4*, and *nad5,* recurrently lack a canonical stop at the homologous position (Figure 4; Supplementary Table S4). *nad4* lacks the reference-position stop in all 10 species, whereas the other three genes vary among species. None of the 19 genomes has a stop immediately after the conserved ENSLIELA motif of *cob*. Stop-free intervals extend for 134 to more than 300 codons in *cox1* of five species and for more than 100 codons in *nad5* of *R. americana*, *R. culbersoniorum*, and *R. intestiniformis*; full states and distances are provided in Supplementary Table S4.

The terminal states were reproduced by both assemblers for the newly sequenced genomes and by reassembly of available public reads, supporting the genomic calls but not the mechanism of translation termination. The affected genes remain consistent with purifying selection: mean pairwise dN/dS values are well below 1, and FEL identifies substantially more negatively than positively selected sites (Supplementary Table S5). Thus, the conserved coding regions show no evidence of generalized relaxation consistent with pseudogenization.

## 4. Discussion

### 4.1. Conserved Core Gene Content and Intron-Associated Genome Size Variation

The 10 *Ramalina* mitogenomes share a stable core gene set but differ 1.83-fold in size, principally in association with intron content. Similar patterns occur in *Cladonia*, *Peltigera*, *Xanthoparmelia*, and broader samples of lichenized fungi [9,10,11]. Recurrent *cox1* and *cob* insertion sites and frequent LAGLIDADG or GIY-YIG domains accord with the established behavior of fungal group I introns [5,6,40,41]. Concentration at homologous sites is compatible with differential loss, recurrent homing, or both, but the present sampling does not distinguish these histories. Nevertheless, the similarly strong association of genome size with HEG number, the near-universal presence of HEG domains in protein-coding introns, and the contrast with the two short, ORF-free *rnl* introns conserved across all ten species are consistent with a major role for homing endonuclease activity in intron turnover. This pattern accords with the recurrent invasion–degeneration–loss cycle proposed for homing endonucleases [41,42], although comparative data alone cannot determine whether HEG activity promotes intron persistence or merely accompanies it. Thus, mitogenome size variation in *Ramalina* is driven primarily by differences in intron content, with a smaller contribution from non-intronic, predominantly intergenic sequence. Whether the intron differences reflect repeated acquisition, differential loss, or both cannot be resolved from the present sampling. Across the sampled *Ramalina* species, intron content, HEG abundance, and genome size vary substantially without a simple clade-specific pattern, suggesting that these traits are evolutionarily labile rather than strongly phylogenetically conserved (Figure 4).

Gene order remains largely conserved, with the *nad6–rns* inversion shared by *R. americana* and *R. culbersoniorum* as the clearest rearrangement. A similar separation of intron or intergenic expansion from gene-order change has been reported in *Tremella fuciformis* and broader fungal comparisons [43,44]. *Ramalina* therefore combines substantial intron-associated size variation with a comparatively stable genomic backbone.

### 4.2. Recurrent Absence of Canonical Stop Codons in Core Respiratory Genes

Canonical stop codons are recurrently absent at conserved terminal positions in *cox1*, *nad3*, *nad4*, and *nad5*, while *cob* lacks an immediate stop after ENSLIELA in all 19 genomes examined. Comparable cases have been reported in lichen-forming fungi, including *nad3* of *Umbilicaria pustulata* [13], and the RefSeq *R. intermedia* record (NC_044483.1) annotates *nad4* and *cob* as 3′-partial. Their restriction to five genes, together with the contrast between the uniform states of *nad4* (all ten species) and *cob* (all 19 genomes) and the species-variable states of *cox1*, *nad3*, and *nad5*, indicates a gene-specific pattern. The uniform *nad4* and *cob* states are consistent with relatively early origins followed by conservation, whereas the variation in *cox1*, *nad3*, and *nad5* indicates greater evolutionary lability within *Ramalina*.

Agreement across independent assemblies and public-read reassemblies, together with full-length conserved coding regions and evidence of purifying selection, supports the genomic terminal states and argues against generalized pseudogenization. This evidence concerns the conserved coding regions and does not directly explain how the terminal states originated or are maintained. Moreover, genomic data do not define mature transcript or protein termini. In budding yeasts, 3′-end mapping showed that RNA processing removes encoded stop codons from mature *nad4* and *nad5* transcripts [45], illustrating that processing can alter the terminal state inferred from genomic sequence. Whether a related or distinct mechanism operates in *Ramalina* requires transcript- or protein-level data.

## 5. Conclusions

This study characterizes four *Ramalina* mitogenomes within a 10-species comparative framework. Genome size varies 1.83-fold in association with intron and HEG content, while four respiratory genes recurrently lack canonical terminal stop codons and cob consistently lacks an immediate stop after the conserved ENSLIELA motif. Together, these results show that mitogenome evolution in *Ramalina* involves both intron-associated genome variation and recurrent, gene-specific changes in mitochondrial gene-terminal states.

## Figures and Tables

**Table 1:** Mitochondrial genomes of the 10 *Ramalina* species comprising the primary comparative dataset. All genomes contain the same 15 core protein-coding genes and two rRNA genes. HEG ORFs are intron-encoded ORFs containing a LAGLIDADG or GIY-YIG homing endonuclease domain. Newly sequenced genomes are in bold.

Species	Accession	Size (bp)	GC (%)	tRNAs	Introns	Intron Content (bp, %)	HEG ORFs
*R. farinacea*	JAPUFD010000030/SRR22733898	29,826	30.44	25	2	437 (1.5)	0
*R. intermedia*	NC_044483.1	30,678	30.34	25	2	436 (1.4)	0
*R. petrina*	BK074022.1	31,578	30.52	25	3	1694 (5.4)	0
*R. sinensis*	OP271861.1	38,265	30.70	25	8	6744 (17.6)	2
*R. culbersoniorum*	BK074021.1	46,296	31.54	25	6	12,084 (26.1)	4
*R. americana*	BK074020.1	46,594	31.86	25	8	12,457 (26.7)	4
*R. siliquosa*	OZ244615.1	48,923	30.80	25	10	12,408 (25.4)	7
***R. yasudae***	**PZ861325**	**49,570**	**30.74**	**26**	**12**	**13,562 (27.4)**	**10**
***R. pacifica***	**PZ861323**	**51,296**	**30.46**	**25**	**13**	**15,139 (29.5)**	**12**
***R. intestiniformis***	**PZ861324**	**54,653**	**30.38**	**26**	**12**	**15,533 (28.4)**	**11**

## Data Availability

The three newly sequenced mitogenomes and their annotations have been deposited in GenBank under accessions PZ861323–PZ861325. Public sequences and raw-read accessions used in the comparative and validation analyses are listed in Supplementary Table S1.

## Supplementary Materials

The following supporting information can be downloaded at: https://www.mdpi.com/article/10.3390/jof12100729/s1, Table S1: Accessions, provenance, and summary statistics of the 19 analyzed mitogenomes and validation reassemblies; Table S2: Complete intron inventory with reference insertion sites, MFannot subtypes, and HEG occupancy; Table S3: RSCU values of the 15 core protein-coding genes; Table S4: Complete stop-codon survey, including validation reassemblies, reference C-termini, and observed stop-free distances; Table S5: Selection statistics for 14 protein-coding genes; Table S6: Repeat-content summary; Figure S1: Synteny and gene-order comparison of the 19 Ramalinaceae mitogenomes, aligned from the *cox1* start and drawn to scale; Figure S2: Nineteen-taxon maximum-likelihood tree based on 14 concatenated protein-coding genes, with *Bacidia* as the outgroup, and terminal states of the five affected genes.
