# Supplementary material for: Intron-Associated Mitogenome Size Variation and Gene-Specific Stop Codon Absence in the Lichen-Forming Genus Ramalina
Source: J Fungi (Basel). 2026 Sep 30;12(10):729. doi: 10.3390/jof12100729 (PMC13706191; doi:10.3390/jof12100729)
Supplement: Supplementary file 1 [file jof-12-00729-s001.zip › Figure S1.pdf]

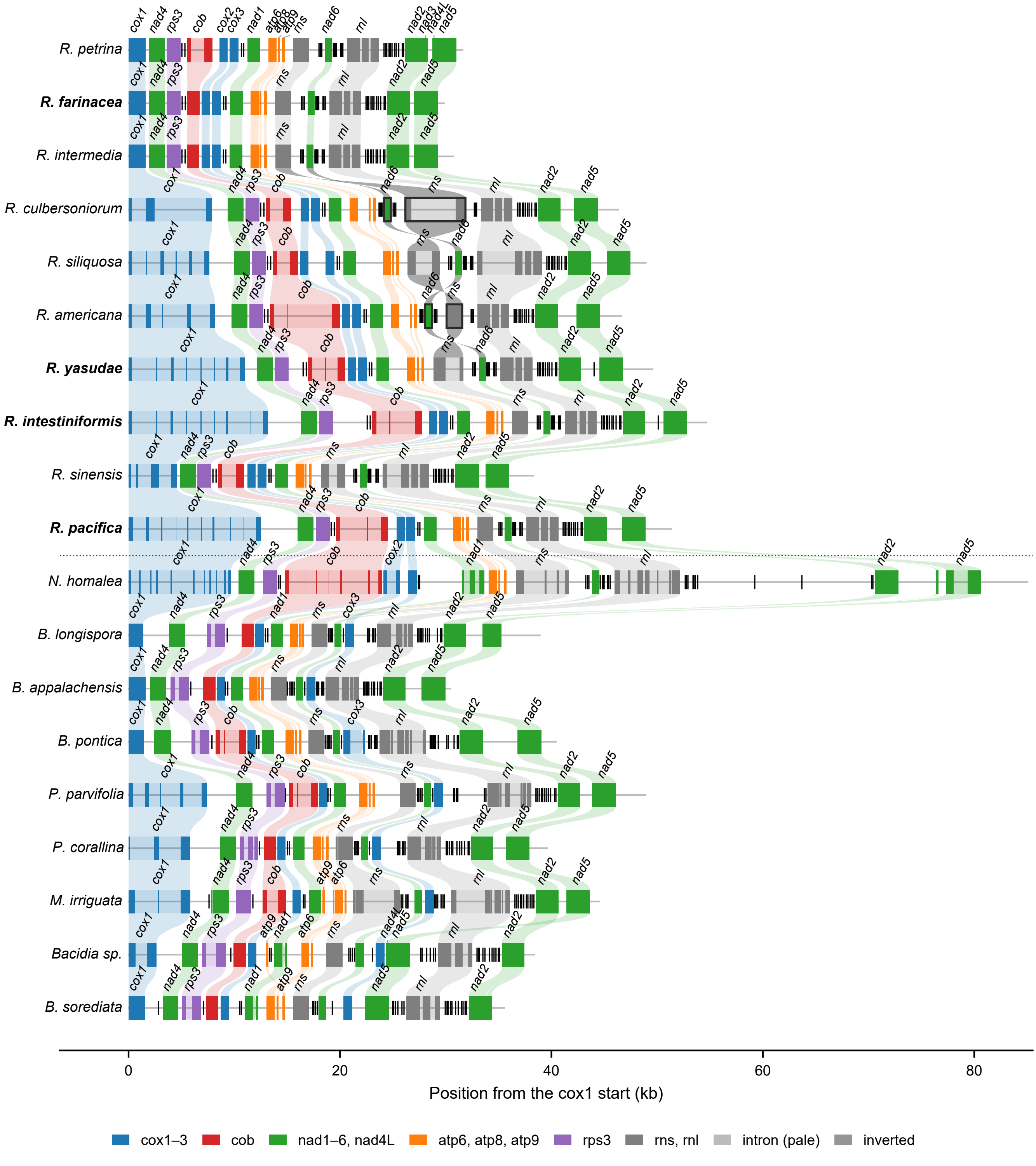
R. petrina
R. farinacea
R. intermedia
R. culbersoniorum
R. siliquosa
R. americana
R. yasudae
R. intestiniformis
R. sinensis
R. pacifica
N. homalea
B. longispora
B. appalachensis
B. pontica
P. parvifolia
P. corallina
M. irriguata
Bacidia sp.
B. sorediata
cox1
nad4
rps3
cob
cox2
cox3
nad1
atp6
atp8
atp9
rns
nad6
rnl
nad2
nad3
nad4L
nad5
0
20
40
60
80
Position from the cox1 start (kb)
cox1–3
cob
nad1–6, nad4L
atp6, atp8, atp9
rps3
rns, rnl
intron (pale)
inverted
