# Supplementary material for: Intron-Associated Mitogenome Size Variation and Gene-Specific Stop Codon Absence in the Lichen-Forming Genus Ramalina
Source: J Fungi (Basel). 2026 Sep 30;12(10):729. doi: 10.3390/jof12100729 (PMC13706191; doi:10.3390/jof12100729)
Supplement: Supplementary file 1 [file jof-12-00729-s001.zip › Figure S2.pdf]

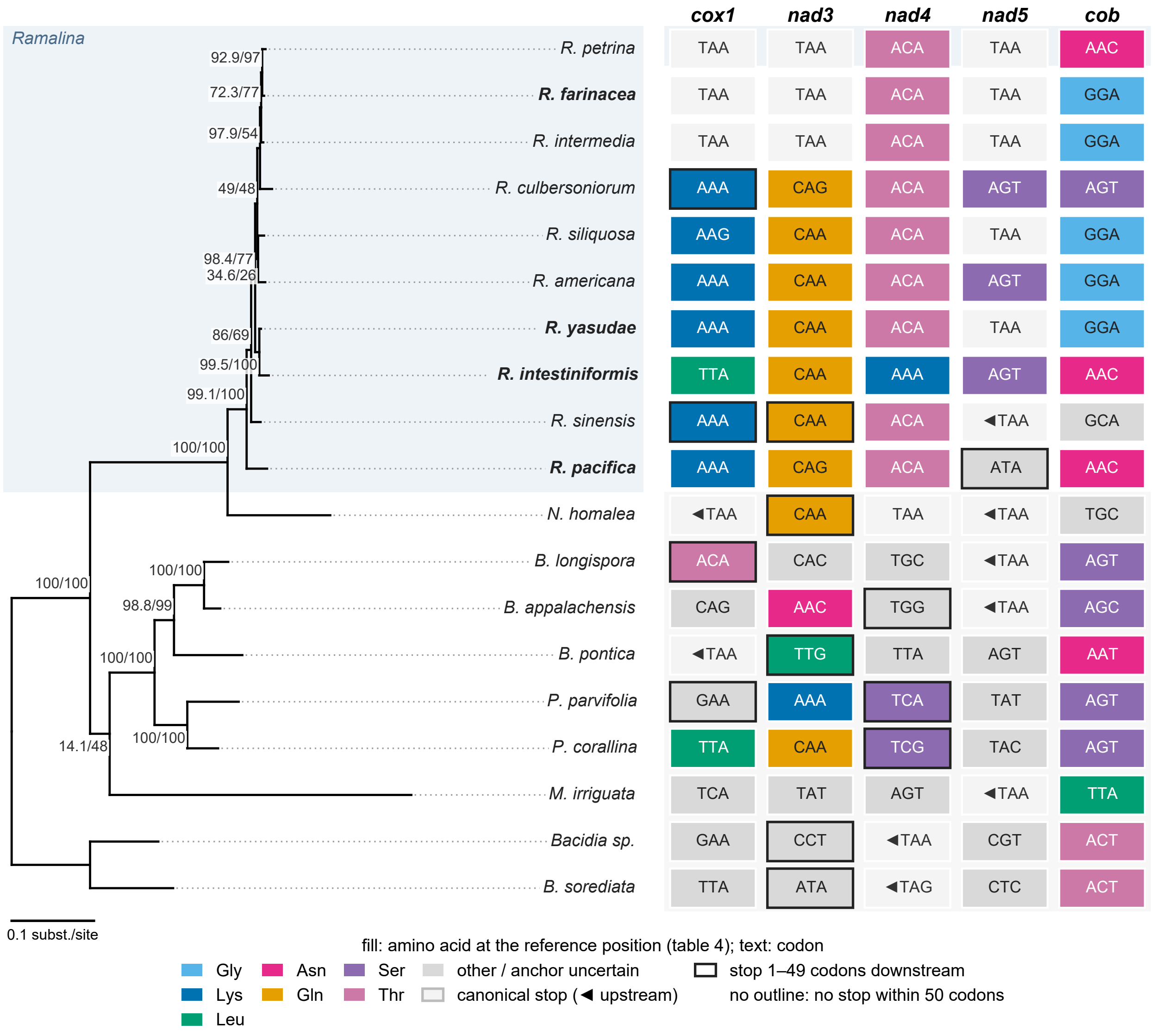
Ramalina
cox1
nad3
nad4
nad5
cob
R. petrina
TAA TAA ACA TAA AAC
92.9/97
R. farinacea
TAA TAA ACA TAA GGA
72.3/77
R. intermedia
TAA TAA ACA TAA GGA
97.9/54
R. culbersoniorum
AAA CAG ACA AGT AGT
49/48
R. siliquosa
AAG CAA ACA TAA GGA
98.4/77
34.6/26
R. americana
AAA CAA ACA AGT GGA
R. yasudae
AAA CAA ACA TAA GGA
86/69
R. intestiniformis
TTA CAA AAA AGT AAC
99.5/100
99.1/100
R. sinensis
AAA CAA ACA ◀TAA GCA
100/100
R. pacifica
AAA CAG ACA ATA AAC
N. homalea
◀TAA CAA TAA ◀TAA TGC
B. longispora
ACA CAC TGC ◀TAA AGT
100/100
100/100
B. appalachensis
CAG AAC TGG ◀TAA AGC
98.8/99
B. pontica
◀TAA TTG TTA AGT AAT
100/100
P. parvifolia
GAA AAA TCA TAT AGT
P. corallina
TTA CAA TCG TAC AGT
14.1/48
100/100
M. irriguata
TCA TAT AGT ◀TAA TTA
Bacidia sp.
GAA CCT ◀TAA CGT ACT
B. sorediata
TTA ATA ◀TAG CTC ACT
0.1 subst./site
fill: amino acid at the reference position (table 4); text: codon
Gly
Lys
Leu
Asn
Gln
Ser
Thr
other / anchor uncertain
canonical stop (◀ upstream)
stop 1–49 codons downstream
no outline: no stop within 50 codons
